# Asiatic Acid Alleviates Hemodynamic and Metabolic Alterations via Restoring eNOS/iNOS Expression, Oxidative Stress, and Inflammation in Diet-Induced Metabolic Syndrome Rats

**DOI:** 10.3390/nu6010355

**Published:** 2014-01-16

**Authors:** Poungrat Pakdeechote, Sarawoot Bunbupha, Upa Kukongviriyapan, Parichat Prachaney, Wilaiwan Khrisanapant, Veerapol Kukongviriyapan

**Affiliations:** 1 Department of Physiology, Faculty of Medicine, Khon Kaen University, Khon Kaen 40002, Thailand; E-Mails: bugvo@hotmail.com (S.B.); upa_ku@kku.ac.th (U.K.); wilkhr@kku.ac.th (W.K.); 2 Department of Anatomy, Faculty of Medicine, Khon Kaen University, Khon Kaen 40002, Thailand; E-Mail: parpra@kku.ac.th; 3 Department of Pharmacology, Faculty of Medicine, Khon Kaen University, Khon Kaen 40002, Thailand; E-Mail: veerapol@kku.ac.th

**Keywords:** metabolic syndrome, asiatic acid, inflammation, oxidative stress

## Abstract

Asiatic acid is a triterpenoid isolated from *Centella asiatica*. The present study aimed to investigate whether asiatic acid could lessen the metabolic, cardiovascular complications in rats with metabolic syndrome (MS) induced by a high-carbohydrate, high-fat (HCHF) diet. Male Sprague-Dawley rats were fed with HCHF diet with 15% fructose in drinking water for 12 weeks to induce MS. MS rats were treated with asiatic acid (10 or 20 mg/kg/day) or vehicle for a further three weeks. MS rats had an impairment of oral glucose tolerance, increases in fasting blood glucose, serum insulin, total cholesterol, triglycerides, mean arterial blood pressure, heart rate, and hindlimb vascular resistance; these were related to the augmentation of vascular superoxide anion production, plasma malondialdehyde and tumor necrosis factor-alpha (TNF-α) levels (*p* < 0.05). Plasma nitrate and nitrite (NOx) were markedly high with upregulation of inducible nitric oxide synthase (iNOS) expression, but dowregulation of endothelial nitric oxide synthase (eNOS) expression (*p* < 0.05). Asiatic acid significantly improved insulin sensitivity, lipid profiles, hemodynamic parameters, oxidative stress markers, plasma TNF-α, NOx, and recovered abnormality of eNOS/iNOS expressions in MS rats (*p* < 0.05). In conclusion, asiatic acid improved metabolic, hemodynamic abnormalities in MS rats that could be associated with its antioxidant, anti-inflammatory effects and recovering regulation of eNOS/iNOS expression.

## 1. Introduction

High-carbohydrate, high-fat (HCHF) diet-induced metabolic and cardiovascular abnormalities in rats that closely mimic characteristics of metabolic syndrome in human have been used in several studies [1,2,3]. Signs of metabolic and cardiovascular alterations shown in metabolic syndrome are central obesity, insulin resistance, impaired glucose tolerance, hypertension, and dyslipidemia [4]. Importantly, metabolic syndrome is an early state of cardiovascular disease and diabetes mellitus which are major causes of serious health problems and increased mortality throughout the world [5,6].

There is evidence to show that an animal model of diet induced-metabolic syndrome is closely linked with oxidative stress [7]. Rats fed with a high-fat diet exhibited metabolic syndrome with an increase in superoxide (O_2_^•−^) production and subsequent formation of peroxynitrite (ONOO^−^) that cause inactivation of nitric oxide (NO) [8]. Furthermore, serum malondialdehyde (MDA), protein carbonyl, C-reactive protein, and xanthine oxidase were increased while superoxide dismutase (SOD) activity was decreased in metabolic syndrome patients [9]. Additionally, chronic inflammation present in diet induced metabolic syndrome rats leading to cardiac and vascular dysfunction has been reported [10]. Nuclear factor-κB (NF-κB) protein levels were enhanced, together with a decrease in protein levels of nuclear factor erythroid-2-related factor-2 (Nrf2) and carnitine palmitoyltransferase 1 (CPT1) in liver and cardiac tissues of high-carbohydrate, high-fat diet-induced metabolic syndrome rats [11]. In addition, in obese humans, a proinflammatory cytokine, TNF-α was found in plasma and served as a marker of insulin resistance [12]. Therefore, it is noteworthy that the oxidative stress and chronic inflammation clearly observed in metabolic syndrome are likely to be important in the development cardiovascular complications including, atherosclerosis and endothelium dysfunction. A later complication that is well-established to involve inactivation of NO bioactivity, as the alterations of endothelial nitric oxide synthase (eNOS) and inducible nitric oxide synthase (iNOS) expression was observed in diet-induced metabolic syndrome rats. This was supported by downregulation of eNOS expression in aorta [13], as well as overexpression of iNOS protein in vascular tissues in diet-induced metabolic syndrome rats [8].

Asiatic acid, a triterpenoid derivative from *Centella asiatica*, has been reported to have biological effects such as antioxidant as well as anti-inflammation. Lee and coworkers (2000) showed the neuroprotective effect of asiatic acid in cell cultures, which was associated with its antioxidant effects [14]. It also reduced H_2_O_2_-related cell death and decreased intracellular free radical concentrations [15]. Furthermore, antinociceptive activities and the mechanisms of anti-inflammation of asiatic acid in mice has been demonstrated that was related to decreases in the level of MDA, iNOS, cyclooxygenase-2 (COX-2), and NF-κB in the edema paw with increasing the activities of catalase, SOD, and glutathione peroxidase (GPx) in the liver [16]. Recently, the anti-diabetic effect of asiatic acid in modulating the key regulatory enzymes has been shown in streptozotocin-induced diabetic rats [17]. Concurrently, the therapeutic effect of asiatic acid on dyslipidemia in streptozotocin-induced diabetic rats has been described. This antihyperlipidemic activity of asiatic acid could prevent atherosclerosis, a major pathogenesis of cardiovascular disease [18]. Although a wide range of potentially therapeutic effect of asiatic acid have been reported, little is known about the effect of asiatic acid on metabolic syndrome associated with hypertension in diet-induced MS. The aim of the present study was to investigate whether asiatic acid can improve metabolic and cardiovascular complications in an animal model of HCHF diet-induced metabolic syndrome.

## 2. Experimental Section

### 2.1. Animals

Adult male Sprague-Dawley rats weighing 180–200 g (six-weeks old) were obtained from the National Laboratory Animal Center, Mahidol University, Salaya, Nakornpathom, Thailand. All animals were housed in the HVAC (Heating, Ventilation and Air-Conditioning) System with 12 h dark/light cycle at the Northeast Laboratory Animal Center, Khon Kaen University, Thailand. All animal procedures were controlled and approved by the Institutional Animal Ethics Committee of Khon Kaen University (AEKKU 36/2555). After one week of acclimatization, the animals were fed with HCHF diet and 15% fructose in drinking water for 12 weeks to induce metabolic syndrome while normal control rats were fed with standard normal diet with normal drinking water. The composition of HCHF diet followed the method of Brown and co-workers [19]. In addition, rat body weight was assessed once a week.

### 2.2. Experimental Protocols

Animals were randomly divided into five groups (*n* = 8–10/group) and were daily treated with asiatic acid or vehicle. Control group: normal rats were intragastrically administered with vehicle (propylene glycol) at 0.15 mL/100 g BW/day for three consecutive weeks. Control rats-treated group: normal rats were intragastrically administered with asiatic acid (20 mg/kg/day) for three consecutive weeks. MS group: MS rats were intragastrically administered with vehicle at 0.15 mL/100 g BW/day for three consecutive weeks. MS-treated group: MS rats were intragastrically administered with asiatic acid (10 or 20 mg/kg/day) for three consecutive weeks. The dose of asiatic acid used in this study is based on the report regarding its anti-diabetic effect in streptozotocin-induced diabetic rats [17].

### 2.3. Indirect Measurement of Blood Pressure in Conscious Rats

Systolic blood pressure (SP) of animals was measured weekly using non-invasive tail-cuff plethysmography (IITC/Life Science Instrument model 229 and model 179 amplifier; Woodland Hills, CA, USA) once a month to assess blood pressure changes throughout the 15 weeks of the study.

### 2.4. Fasting Blood Glucose (FBG), Oral Glucose Tolerance Test (OGTT), and Serum Insulin Assessments

The FBG was investigated once a month in all groups of rats. Rats were fasted overnight (8–10 h) and blood samples were taken from a lateral tail vein to measure the FBG using a glucometer (Roche Diagnostics Australia Pty. Ltd., Sydney, Australia). Then, the animals were orally administered with glucose at a dose of 2 g/kg BW in order to determine glucose tolerance. The blood glucose concentration before glucose loading (0 min) and at 30, 60, 120, and 150 min after glucose administration was investigated. Animals that have significantly higher blood glucose at 150 min than at 0 min were assumed to have an impaired glucose tolerance and were included in the experiment as a MS group.

### 2.5. Fasting Serum Insulin Assessments and HOMA-IR Calculation

The fasting serum insulin concentration (basal secretion) at the end of three weeks of treatment was measured using a Rat Insulin ELISA Kit (Millipore Corporation, Billerica, MA, USA). HOMA-IR score was used as an index of insulin resistance [20] and calculated using Equation 1:

HOMA = (fasting glucose (mmol/L) × fasting insulin (μIU/mL))/22.5
(1)

### 2.6. Hemodynamic Measurements

At the end of this study, rats were anesthetized by peritoneal injection of pentobarbital-sodium (60 mg/kg) and placed on a heating pad. A tracheotomy was made to assist respiration. The femoral artery was cannulated with a polyethylene tube. SP, diastolic blood pressure (DP), mean arterial pressure (MAP), and heart rate (HR) were continuously monitored by way of pressure transducers and recorded using the Acknowledge Data Acquisition and Analysis Software (Biopac Systems, Santa Barbara, CA, USA). The abdominal aorta was carefully separated from the abdominal vein, cleaned of connective tissues and fitted with a flow probe to detect hind limb blood flow (HBF) with an electromagnetic flowmeter (Carolina Medical Electronics, Carolina, NC, USA). Hind limb vascular resistance (HVR) was calculated by MAP divided by HBF. Blood samples were collected via the abdominal aorta for biochemical assays and liver function test. Carotid arteries (about 2 cm in length) were cut out rapidly from animals for assessing superoxide production. The aorta was collected to measure eNOS and iNOS expression. Heart, left ventricular (LV), and liver wet weight were measured.

### 2.7. Measurement of Oxidative Stress Markers

#### 2.7.1. O_2_^•−^ Production

Production of O_2_^•−^ in vascular tissues was determined by lucigenin-enhanced chemiluminescence as previously described, with some modification [21]. The carotid artery was excised rapidly, placed in ice-cold saline. Vessel segments were cut into 1 cm lengths and incubated with 1 mL oxygenated Krebs-Ringer bicarbonate solution at pH 7.4, 37 °C, for 30 min. Lucigenin 100 μM was added in sample tubes and placed in a luminometer (Turner Biosystems, Sunnyvale, CA, USA). Luminometer counts were integrated every 30 s for 5 min and averaged. The vessels were dried at 45 °C for 24 h and weighed. O_2_^•−^ production was expressed as relative light unit count/mg dry wt/min.

#### 2.7.2. Plasma MDA

The levels of MDA were assayed following a previous described method of Nakmareong and coworkers (2011) [22]. In brief, 150 μL of plasma was reacted with 10% TCA, 125 μL of 5 mM ethylenediamine tetraacetic acid (EDTA), 125 μL of 8% sodium dodecylsulfate (SDS), and 10 μL of 0.5 μL/mL of butylated hydroxyluene (BHT). The mixture was left for 10 min, then 0.6% thiobarbituric acid (TBA) was added in an equal volume and the mixture was heated for 30 min in a boiling water bath. After cooling to room temperature, the mixture was centrifuged 10,000× *g* for 5 min at 25 °C. The absorbance of the supernatant was measured at 532 nm by a spectrophotometer (Amersham Bioscience, Arlington, MA, USA).

#### 2.7.3. Plasma NOx

Nitrate and nitrite, the end products of NO metabolism, were used as an indicator of NOS activity. Plasma NOx was measured using an enzymatic conversion method with Griess reaction [22]. In brief, plasma samples were deprotenized by using ultrafiltration (Pall Corp., Ann Arbor, MI, USA), treated with converting enzymes and then reacted with a Griess solution. The absorbance of samples was measured on an enzyme-linked immunosorbent assay (ELISA) plate reader with a filter wavelength of 540 nm (Tecan GmbH., Grodig, Australia).

#### 2.7.4. TNF-α

The concentration of plasma TNF-α was measured using an enzyme-immunoassay (ELISA) kit (eBioscienc, Inc., San Diego, CA, USA).

### 2.8. Western Blot Analysis

eNOS and iNOS expression in the aortic homogenates were determined [22] with some modifications. Protein of homogenates was run on a sodium dodecyl sulfate polyacrylamide gel electrophoresis system. The proteins were electrotransferred onto a polyvinylidene difluoride (PVDF) membrane, blocked with 5% skimmed milk in phosphate buffer saline with 0.1% Tween-20 (PBST) for 2 h and incubated overnight with primary antibody against eNOS (1:1000) (BD Biosciences, Qume Drive, San Jose, CA, USA) and iNOS (1:1000) (Santa Cruz Biotechnology, Indian Gulch, CA, USA). After washing, the membrane was incubated for 2 h at room temperature with the appropriate horseradish peroxidase secondary antibody conjugated (Santa Cruz Biotechnology, Indian Gulch, CA, USA) to detect bands with Amersham™ ECL™ Prime (Amersham Biosciences Corp., Piscataway, NJ, USA). The densitometric analysis was performed using ImageQuant™ 400 (GE Healthcare Life science, Piscataway, NJ, USA). The intensity of the bands was normalized to that of β-actin and data was expressed as a percentage of the values in control aorta from the same gel.

### 2.9. Chemicals

**Figure 1:**
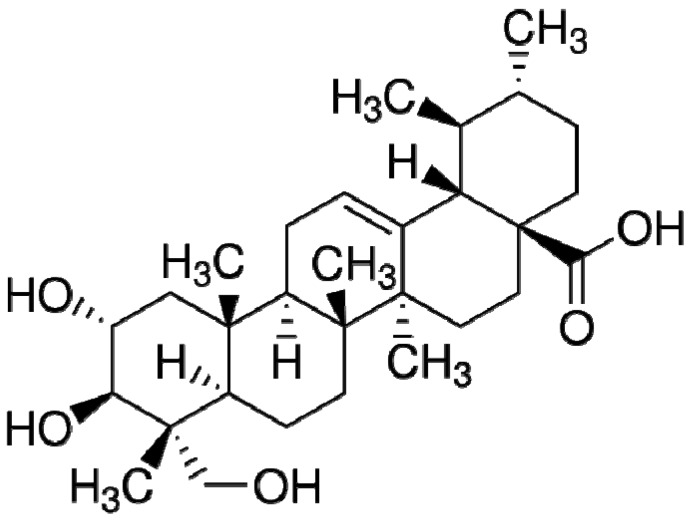
Chemical structure of asiatic acid (from Sigma-Aldrich).

Asiatic acid (Figure 1) (purity > 95%), l-NAME, ethylenediamine tetraacetic acid (EDTA), thiobarbituric acid (TBA), sodium dodecylsulfate (SDS), butylated hydroxyluene (BHT), *N*-1 nepthylethylenediamine dihydrochloride (NED) and sulfanilamide were obtained from Sigma-Aldrich Corp. (St Louis, MO, USA). Nitrate reductase, nicotinamide adenine dinucleotide phosphate (NADPH), glucose-6-phosphate disodium and glucose-6-phosphate dehydrogenase were obtained from Roche Applied Sciences (Mannheim, Germany). Trichloroacetic acid (TCA) and lucigenin were obtained from Fluka Chemika Co. Ltd. (Buch, Switzerland). All other chemicals used were of analytical grade quality.

### 2.10. Statistical Analysis

Data are expressed as means ± standard error of mean (SEM). Statistical comparisons between groups were made using one-way analysis of variance (ANOVA) with a Student Newman–Keul’s test. All analysis was performed using Stata10.1 software (StataCorp, College Station, TX, USA). Statistical significance was determined at a level of *p* < 0.05.

## 3. Results

### 3.1. Effects of the Asiatic Acid on Metabolic Abnormalities

Significant increases in FBG, fasting serum insulin and HOMA-IR scores were found in rats fed with HCHF diet (*p* < 0.05). Treatment with asiatic acid (10 or 20 mg/kg) for three weeks significantly reduced FBG, fasting serum insulin and HOMA-IR scores compared to those of MS rats without treatment (*p* < 0.05). A decrease of insulin resistance was more pronounced in 20 mg/kg of asiatic acid treatment in MS rats. The AUC of glucose concentration during OGTT was higher in MS rats than in rats fed with a normal diet, indicating glucose intolerance in this group (*p* < 0.05). This impairment of glucose tolerance was significantly recovered by asiatic acid (10 and 20 mg/kg) treatment (*p* < 0.05). Levels of serum total cholesterol and triglycerides were significantly elevated, while serum HDL-C were markedly low in the MS rats compared to those of control group (*p* < 0.05). Administration of asiatic acid (10 or 20 mg/kg) significantly alleviated dyslipidemia by decreasing serum total cholesterol and triglycerides and increasing serum HDL-C in MS rats (*p* < 0.05). In addition, the anti-dyslipidemic activity of asiatic acid in MS rats was dose-dependent. Liver wet weight was significantly increased in MS rats compared to the control group (*p* < 0.05) while there was no different of liver wet weight between MS rats treated with asiatic acid and control group. There were signs of liver dysfunction in MS rats as increased ALT and ALP together with increased liver weight were observed in MS rats (Table 1). However, rats treated with asiatic acid showed improvement of liver function in MS rats as all altered parameters were normalized to normal control levels. This indicates the therapeutic effect of asiatic acid on liver dysfunction in MS rats. There was no significant difference of body weight between MS and control groups. Asiatic acid had no effect on metabolic parameters, liver enzymes and organ weight in normal control rats (Table 1).

**Table 1:** Fasting blood glucose, biochemical parameters, body and organ weight in all experimental groups. Results are expressed as mean ± SEM. AA represents asiatic acid. * *p* < 0.05 *vs*. control group, ^#^ *p* < 0.05 *vs*. MS group, ^†^ *p* < 0.05 *vs*. MS + AA 10 mg/kg group (*n* = 8–10/group).

Parameters	Control	Control + AA 20 mg/kg	MS	MS + AA 10 mg/kg	MS + AA 20 mg/kg
**Fasting blood glucose (mg/dL)**	81.13 ± 1.3	82.88 ± 2.1	116.71 ± 2.8 *	93.50 ± 2.1 ^#^	89.25 ± 1.5 ^#^
**Fasting serum insulin (ng/mL)**	0.45 ± 0.06	0.47 ± 0.06	4.34 ± 0.43 *	2.19 ± 0.13 *^,#^	1.38 ± 0.24 *^,#^^,†^
**AUC (mg/dL/120 min)**	13,065 ± 425	13,018 ± 410	16,125 ± 202 *	14,393 ± 173 ^#^	14,073 ± 180 ^#^
**HOMA-IR score**	2.27 ± 0.32	2.90 ± 0.4	28.90 ± 3.60 *	12.54 ± 0.78 *^,#^	6.72 ± 1.22 *^,#^^,†^
**Cholesterol (mg/dL)**	54.00 ± 2.29	55.38 ± 2.31	89.00 ± 2.31 *	67.38 ± 2.20 *^,#^	61.43 ± 3.26 ^#^
**Triglycerides (mg/dL)**	24.50 ± 3.08	25.75 ± 2.40	70.71 ± 2.71 *	41.71 ± 2.10 *^,#^	29.57 ± 2.27 *^,#^^,†^
**HDL-C (mg/dL)**	41.38 ± 1.29	41.29 ± 1.27	14.86 ± 1.52 *	26.50 ± 0.63 *^,#^	34.13 ± 0.55 *^,#^^,†^
**ALP (U/L)**	73.1 ± 4.5	74.5 ± 3.6	150.7 ± 1.1 *	119.0 ± 4.2 *^,#^	82.2 ± 3.9 ^#^^,†^
**ALT (U/L)**	21.0 ± 0.9	19.2 ± 1.6	34.7 ± 2.6 *	25.0 ± 1.2 *^,#^	21.5 ± 0.8 ^#^
**Body weight (g)**	426.57 ± 5.0	416.75 ± 5.8	423.71 ± 6.5	408.43 ± 6.9	404.86 ± 10.6
**Liver wet weight/body weight (mg/g)**	30.3 ± 1.1	30.7 ± 0.6	43.3 ± 1.2 *	33.6 ± 0.4 *^,#^	32.2 ± 0.3 ^#^^,†^
**Heart wet weight/body weight (mg/g)**	3.37 ± 0.05	3.33 ± 0.03	3.52 ± 0.07	3.39 ± 0.07	3.33 ± 0.1
**LV wet weight/body weight (mg/g)**	2.21 ± 0.02	2.24 ± 0.04	2.34 ± 0.04	2.22 ± 0.03	2.21 ± 0.04

### 3.2. Effects of Asiatic Acid on SP (Indirect Measurement of Blood Pressure)

**Figure 2:**
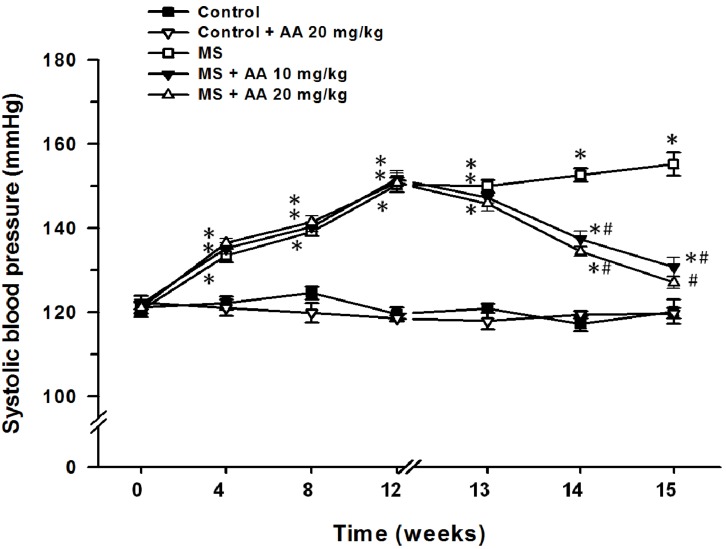
Effect of asiatic acid on systolic blood pressure in all experimental groups. Results are expressed as mean ± SEM. AA represents asiatic acid. * *p* < 0.05 *vs*. control group, ^#^ *p* < 0.05 *vs*. MS group (*n* = 8/group).

Rats received HCHF diet for 12 weeks gradually developed hypertension (SP = 150.3 ± 1.8 mmHg) compared to those of normal control rats (119.6 ± 1.5 mmHg) (*p* < 0.05). However, treatment with asiatic acid (10 and 20 mg/kg) for three weeks significantly attenuated SP (133.6 ± 1.3 mmHg and 127.1 ± 1.5 mmHg, respectively) in MS rats, compared to those of MS rats without treatment (155.2 ± 2.7 mmHg) (*p* < 0.05) (Figure 2).

### 3.3. Effects of Asiatic Acid on Hemodynamic Parameters

HCHF diet-induced mild hypertension, indicated by the significant increases in SP, DP and MAP in the MS group (*p* < 0.05). There was also an increased HR and HVR and a decreased HBF. Furthermore, MS rats treated with asiatic acid (10 or 20 mg/kg) for three weeks had markedly reduced blood pressure, HR and HVR, and increased HBF (*p* < 0.05). Asiatic acid had no effect on hemodynamic parameters in normal control rats (Table 2).

**Table 2:** Changes in hemodynamic parameters in all experimental groups. Results are expressed as mean ± SEM. AA represents asiatic acid. * *p* < 0.05 *vs*. control group, ^#^ *p* < 0.05 *vs*. MS group (*n* = 8/group).

Parameters	Control	Control + AA 20 mg/kg	MS	MS + AA 10 mg/kg	MS + AA 20 mg/kg
**SP (mmHg)**	117.83 ± 1.9	116.61 ± 1.6	149.14 ± 3.6 *	132.91 ± 1.0 *^,#^	128.21 ± 1.7 *^,#^
**DP (mmHg)**	76.96 ± 1.7	77.07 ± 1.5	98.51 ± 2.4 *	88.47 ± 1.5 *^,#^	88.28 ± 1.3 *^,#^
**MAP (mmHg)**	90.58 ± 1.7	90.25 ± 1.4	116.05 ± 2.7 *	103.08 ± 1.1 *^,#^	101.59 ± 1.3 *^,#^
**HR (beat/min)**	345.89 ± 8.6	344.76 ± 5.7	417.32 ± 14.6 *	359.34 ± 4.9 ^#^	356.14 ± 6.4 ^#^
**HBF (mL/min/100 g tissue)**	7.38 ± 0.35	7.49 ± 0.40	4.00 ± 0.18 *	5.91 ± 0.28 *^,#^	6.33 ± 0.29 ^#^
**HVR(mmHg/mL/min/100 g tissue)**	12.44 ± 0.53	12.31 ± 0.73	29.42 ± 1.77 *	17.73 ± 0.84 *^,#^	16.27 ± 0.74 *^,#^

### 3.4. Effects of the Asiatic Acid on Oxidative Stress Markers

**Figure 3:**
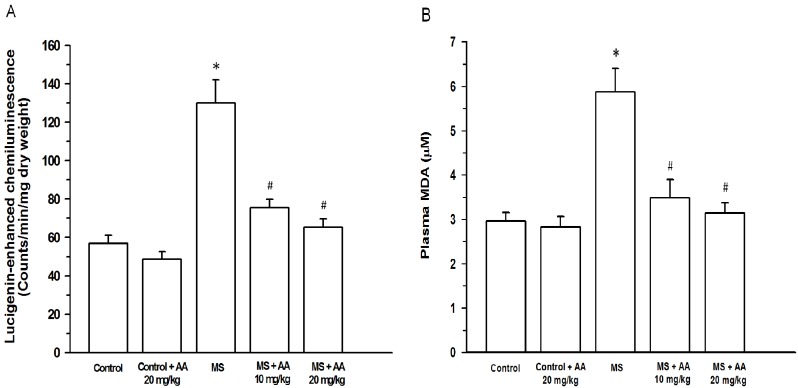
Effect of asiatic acid on vascular superoxide production (**A**) and plasma MDA (**B**) in all experimental groups. Results are expressed as mean ± SEM. AA represents asiatic acid. * *p* < 0.05 *vs*. control group, ^#^ *p* < 0.05 *vs*. MS group (*n* = 8/group).

Increased O_2_^•−^ production in carotid arteries was found in MS rats (130.0 ± 12.0 count/mg dry wt/min) compared to that of the control group (56.9 ± 4.3 count/mg dry wt/min) (*p* < 0.05). Lipid peroxidation occurred in MS rats because there was a significant increase in plasma MDA levels (MS group: 5.9 ± 0.5 μM *vs*. control group: 2.96 ± 0.2 μM, (*p* < 0.05)). Asiatic acid (10 or 20 mg/kg) clearly attenuated O_2_^•−^ production in carotid arteries (75.5 ± 4.4 count/mg dry wt/min and 65.3 ± 4.5 count/mg dry wt/min, respectively) and plasma MDA (3.5 ± 0.4 μM and 3.2 ± 0.2 μM, respectively) (*p* < 0.05) (Figure 3A,B).

### 3.5. Effects of Asiatic Acid on Plasma NOx and TNF-α Concentration

**Figure 4:**
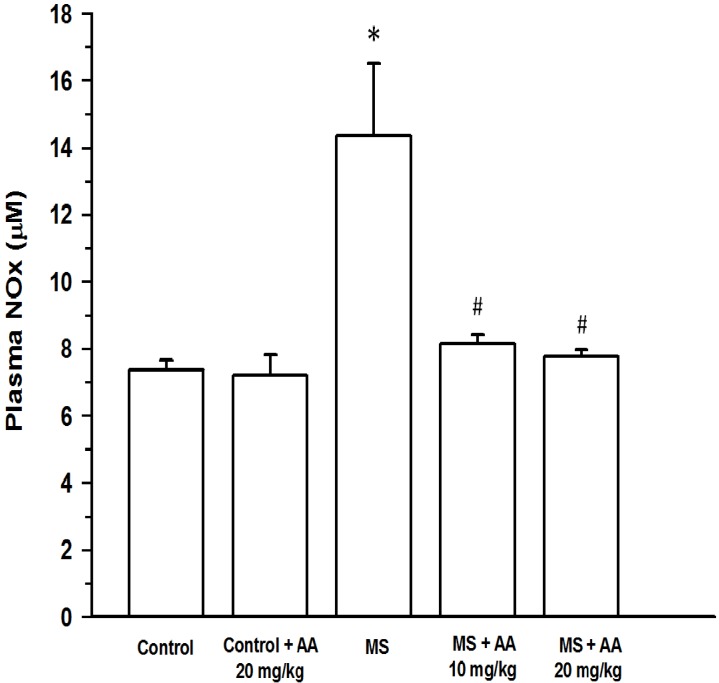
Effect of asiatic acid on plasma nitric oxide metabolites (NOx) in all experimental groups. Results are expressed as mean ± SEM. AA represents asiatic acid. * *p* < 0.05 *vs*. control group, ^#^ *p* < 0.05 *vs*. MS group (*n* = 8/group).

**Figure 5:**
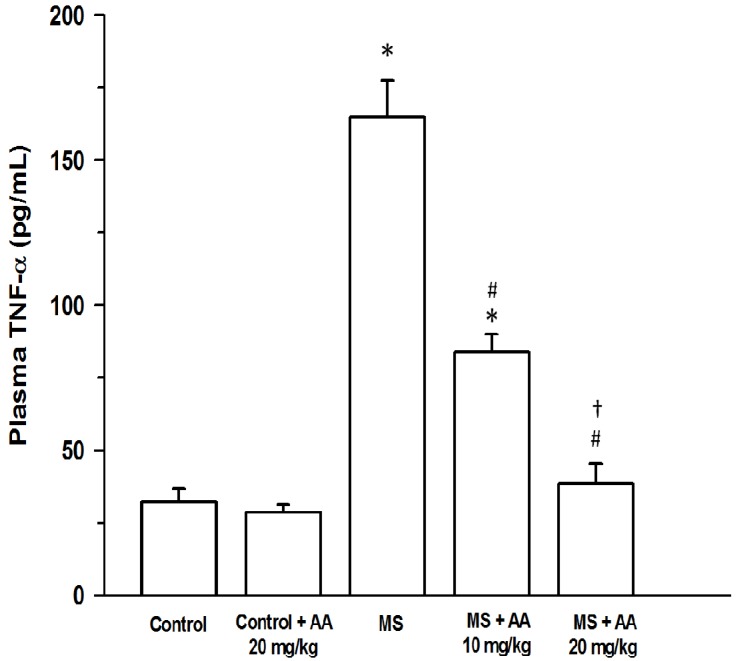
Effect of asiatic acid on plasma TNF-α in all experimental groups. Results are expressed as mean ± SEM. AA represents asiatic acid. * *p* < 0.05 *vs*. control group, ^#^ *p* < 0.05 *vs*. MS group, ^†^ *p* < 0.05 *vs*. MS + AA 10 mg/kg group (*n* = 8/group).

Plasma NOx concentration was increased in MS rats (14.4 ± 2.1 μM) compared to normal rats (7.4 ± 0.3 μM) (*p* < 0.001). Treatment with asiatic acid (10 or 20 mg/kg) led to a significant decrease in plasma NOx (8.1 ± 0.3 μM and 7.8 ± 0.2 μM, respectively) (*p* < 0.05) (Figure 4). An increase in plasma TNF-α levels was found in the MS rats (MS rats: 164.97 ± 12.4 pg/mL *vs*. control rats: 32.4 ± 4.4 pg/mL) (*p* < 0.001). An elevation of plasma TNF-α level in MS rats was attenuated by asiatic acid treatment. Moreover, plasma TNF-α level in MS rats receiving (20 mg/kg) asiatic acid was lower than those of in MS rats treated with (10 mg/kg) asiatic acid (54.9 ± 9.5 pg/mL *vs*.77.8 ± 7.9 pg/mL, (*p* < 0.05)) (Figure 5).

### 3.6. Effects of Asiatic Acid on Expression of iNOS and eNOS Protein in Aortic Tissues

**Figure 6:**
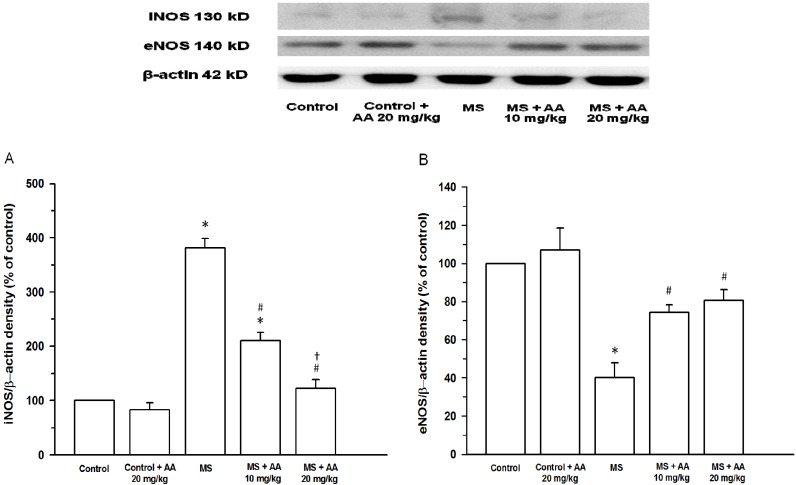
Effect of asiatic acid on iNOS protein expression (**A**) and eNOS protein expression (**B**) in all experimental groups. Results are expressed as mean ± SEM. AA represents asiatic acid. * *p* < 0.05 *vs*. control group, ^#^ *p* < 0.05 *vs*. MS group, ^†^ *p* < 0.05 *vs*. MS + AA 10 mg/kg group (*n* = 4/group).

An upregulation of iNOS expression in aortic tissues was observed in the MS rats (*n* = 4, *p* < 0.001) (Figure 6A). Asiatic acid (10 and 20 mg/kg) suppressed HCHF induced-iNOS expression in aortic tissues. Concurrently, downregulation of eNOS protein expression was also found in MS rats (*p* < 0.05) (Figure 6B). Similarly, treatment with asiatic acid (10 and 20 mg/kg) completely restored aortic eNOS protein expression (*p* < 0.05). The effect of asiatic acid on iNOS and eNOS expression in MS rats was not different between doses.

## 4. Discussion

The present findings show that rats fed with HCHF diet exhibited metabolic syndrome, manifested by hypertension, insulin resistance, hyperglycemia and dyslipidemia. Additionally, inflammation, oxidative stress, increased plasma NOx, downregulation of eNOS, and upregulation of iNOS expression were also observed. We firstly demonstrated that asiatic acid alleviated most of these metabolic defects as it had anti-hypertensive, anti-dyslipidemic effects, improved insulin sensitivity, and antiinflammation. The underlying mechanism may involve asiatic acid decreasing oxidative stress, inflammatory markers as well as restoring eNOS/iNOS expression.

We have shown that asiatic acid reduced FBG, serum insulin, AUC of glucose tolerance as well as HOMAR-IR score, indicating that asiatic acid improved insulin sensitivity and hyperglycemia in HCHF diet-induced MS rats. Subsequently, dyslipidemia was attenuated with asiatic acid treatment in MS rats. Asiatic acid ameliorated these metabolic defects in MS rats were dose-dependent, with the higher dose of asiatic acid being more pronounced. Therefore, asiatic acid at 20 mg/kg is an effective dose to lesson MS. There is evidence to show that asiatic acid significantly reduced blood glucose levels in streptozocin induced-diabetes rats where asiatic acid preserved beta cell death and promoted their proliferation [23]. The underlying mechanism of asiatic acid improving glucose metabolic changes in the present study was unclear. It might be related to its anti-oxidative stress action since several studies have proposed a connection between antioxidant compounds and metabolic syndrome. For example, green tea beverage with high catechins can lower biomarkers of oxidative stress as well as improve features of metabolic syndrome in obese patients [24]. Apocynin, an inhibitor of NADPH oxidase, attenuated hepatic MDA, as well as enhanced antioxidative defense system leading to improvements of insulin sensitivity, blood glucose levels and lipid profiles in high-fat diet mice [25]. Increased ALT and ALP together with increased liver weight in MS rats indicated liver damages. This result was consistent with the study in HCHF diet-induced MS rats [26]. However, increased liver wet weight and impairment of liver function in MS rats were reversed by treatment with asiatic acid in this study. This may be due to asiatic acid reducing lipid peroxidation in liver [27].

Based on the basic cardiovascular physiology, systemic blood pressure is determined by cardiac output and total peripheral resistance. Heart rate and stroke volume modulate cardiac output while TPR is regulated by the diameter of small arteries and arterioles. In the present study, cardiovascular complications induced by the HCHF diet were characterized by high HR, HVR, and low HBF, resulting in hypertension. These hemodynamic changes were normalized in MS rats treated with asiatic acid. In addition, asiatic acid at 10 and 20 mg/kg produced similar effects on cardiovascular changes. The mechanism by which asiatic acid reversed hemodynamic alterations observed in HCHF diet induced MS rats was most likely due to its antiinflammatory and antioxidative effects leading to reduce production of reactive oxygen species and inflammatory cytokines, with increased nitric oxide bioavailability. In addition, it has been reported that *Centella asiatica* extract, a major source of asiatic acid, had angiotensin converting enzyme (ACE) inhibitor effect [28]. It is well established that there are several causes of HCHF and high fructose diet-induced hypertension, such as endothelial dysfunction together with oxidative stress, inflammation [29], increased sympathetic nervous activity [30]. Especially, excessive production of reactive oxygen species in metabolic syndrome has been shown to provoke hypertension as well as cardiovascular disease [31].

O_2_^•−^ production and MDA are oxidative stress markers, which indicated oxidative damage and lipid peroxidation, which can instigate the risk factors in MS rats. Our results showed the high levels of vascular O_2_^•−^ production and plasma MDA in HCHF diet-induced MS rats. These results are consistent with other findings that oxidative stress may be an important characteristic of diet induced-MS in animal models [32] and also in humans [33]. The major source of vascular O_2_^•−^ production in diet-induced metabolic syndrome is probably from NADPH oxidase. This was supported by several studies that there was a significant upregulation of NADPH oxidase subunit in aorta of a rat model of metabolic syndrome [7,34]. It was unlikely that enhanced O_2_^•−^ generation is from NOS uncoupling in MS rats. This is consistent with a huge production of nitric oxide and together with increased iNOS activity found in MS rats. Increased lipid peroxidation and O_2_^•−^ production in carotid arteries were restored closely back to the control level after asiatic acid administration, indicating that asiatic acid exhibited strong antioxidant effect. The antioxidant activity of asiatic acid was supported in ethanol-induced hepatic injury where asiatic acid suppressed oxidative stress and attenuated hepatic damage [27]. There is a study supporting that antioxidant compounds have been shown to alleviate insulin resistance and lipid profile in metabolic diseases. For example, antioxidant substances in the leaf extract from *Passiflora nitida* Kunth decreased plasma glucose in diabetic rats that was associated with a decrease in lipid peroxdation [35].

The high level of circulating TNF-α was primarily considered to be an inflammatory response, which is known to be causally related to insulin resistance and metabolic syndrome state [36,37]. The source of plasma TNF-α found in this study may be accumulative fats and infiltration monocytes [38]. In rats fed with HCHF diet, an elevation of plasma TNF-α was demonstrated concomitantly with upregulation of proinflammatory iNOS protein expression. However, downregulation of eNOS expression in aortic tissues was present in MS rats while total NO production was dramatically increased. Thus, the source of plasma NOx observed in the present study probably reflected by activation by iNOS activity. Under normal physiological conditions, a major source of NO in the circulation is from eNOS in vascular endothelium. A substantial production of NO has been shown to be induced by inflammation, where inflammatory cytokines such as TNF-α, IL-1β, and interferon-γ stimulate iNOS activity [39] and may be responsible for hypertension and insulin.

It has been know that there is the strong relationship between inflammatory responses and metabolic syndrome as inflammation led to insulin resistance and MS in high-fat/high-sugar diet induced MS rats [40]. Additional evidence in human with metabolic defects showed high levels of inflammatory markers [41,42]. In this study, we have provided direct evidence that asiatic acid can alleviate metabolic abnormality supporting by a reduction of plasma TNF-α, an inflammatory markers, in MS rats. This was accompanied by normalization of eNOS and iNOS expression and plasma NOx levels. Several studies reported the anti-inflammatory effect of asiatic acid both *in vivo* and *in vitro*. For example, asiatic acid is able to decrease TNF-α, and interleukin-1β (IL-1β) levels in the λ-carrageenan-induced edema paw in mice [16]. *In vivo*, asiatic acid exhibited an anti-inflammatory effect with being decreased iNOS, COX-2, IL-6, IL-1β, and TNF-α expressions in RAW 264.7 cells [43].

## 5. Conclusions

In summary, we found that asiatic acid improved metabolic and hemodynamic abnormalities in HCHF diet-induced metabolic syndrome in rats by decreasing oxidative stress and inflammation. Additionally, asiatic acid also reduced an excessive NO production that was consistent with a reduction of iNOS activity in MS rats.
